# Supplementary material for: Oral health outcomes and shift working among male workers: A cross-sectional survey
Source: PLoS One. 2022 Oct 17;17(10):e0275924. doi: 10.1371/journal.pone.0275924 (PMC9576084; doi:10.1371/journal.pone.0275924)
Supplement: Attachment — Submitted filename: Comments PONE-D-22-05469.pdf [file pone.0275924.s002.pdf]

PLOS ONE

Oral health and occupational condition in workers of a cement factory

--Manuscript Draft--

**Manuscript Number:**	PONE-D-22-05469
**Article Type:**	Research Article
**Full Title:**	Oral health and occupational condition in workers of a cement factory
**Short Title:**	Oral health & occupational condition
**Corresponding Author:**	Hadi Ghasemi<br>Shahid Beheshti University of Medical Sciences<br>IRAN, ISLAMIC REPUBLIC OF
**Keywords:**	Shift work schedule, oral health, occupational stress, DMF index, periodontal index
**Abstract:**	Background and aim: Working time outside routine daily working hours is known as shift working. Studies have shown adverse effects of shift working such as stress, obesity, and diabetes on the workers' health. The aim of the present study is to evaluate the association between oral health and occupational condition in shift workers and non-shift workers of a cement factory in Shahroud, Iran.<br>Methods: Study population comprised 180 male workers of Shahrood cement factory in the year 2015. Convenience sampling was used to select the subjects and continued until completing 90 in each group of shift- and non-shift workers. Data collection included WHO standard oral health questionnaire, health and safety executive (HSE) questionnaires, together with clinical oral examination. The Chi-square test, Pearson correlation coefficient, and generalized Poisson model were employed for statistical evaluation.<br>Results: Mean age of the workers was 39.19 (±9.48); 53% had educational level of less than diploma. Their mean DMFT was 12.89 (±5.75) which correlated with number of years in shift work schedule (Pearson correlation coefficient: 0.41; $p<0.001$) but not correlated with job stress (Pearson correlation coefficient: -0.11; $p=0.12$). Mean number of deep periodontal pockets among the workers was 5.03 (±1.84) that showed correlation with number of years in shift work schedule (Pearson correlation coefficient: 0.33; $p<0.001$) but no correlation with job stress (Pearson correlation coefficient: -0.03; $p=0.68$). Adherence to various oral health behaviors was reported by less than half of the workers.<br>Conclusion: Positive correlation between the prevalence of two main oral diseases and shift working partly signals negative impact of working conditions on oral health among this group of workers. This calls for oral health promotion programs with modifications in their working environment to facilitate health practices.
**Order of Authors:**	Hadi Ghasemi
	Reza Darmohammadi
	Mahshid Namdari
	Zahra Ghorbani
**Additional Information:**	
**Question**	**Response**
**Financial Disclosure**<br><br>Enter a financial disclosure statement that describes the sources of funding for the work included in this submission. Review the submission guidelines for detailed requirements. View published research articles from *PLOS ONE* for specific examples.	The author(s) received no specific funding for this work.

Powered by Editorial Manager® and ProduXion Manager® from Aries Systems Corporation

This statement is required for submission and **will appear in the published article** if the submission is accepted. Please make sure it is accurate.<br><br>**Unfunded studies**<br>Enter: *The author(s) received no specific funding for this work.*<br><br>**Funded studies**<br>Enter a statement with the following details:<br>• Initials of the authors who received each award<br>• Grant numbers awarded to each author<br>• The full name of each funder<br>• URL of each funder website<br>• Did the sponsors or funders play any role in the study design, data collection and analysis, decision to publish, or preparation of the manuscript?<br>• **NO** - Include this sentence at the end of your statement: *The funders had no role in study design, data collection and analysis, decision to publish, or preparation of the manuscript.*<br>• **YES** - Specify the role(s) played.<br><br>* typeset	
**Competing Interests**<br><br>Use the instructions below to enter a competing interest statement for this submission. On behalf of all authors, disclose any competing interests that could be perceived to bias this work—acknowledging all financial support and any other relevant financial or non-financial competing interests.<br><br>This statement is required for submission and **will appear in the published article** if the submission is accepted. Please make sure it is accurate and that any funding sources listed in your Funding Information later in the submission form are also declared in your Financial Disclosure statement.<br><br>View published research articles from	The authors have declared that no competing interests exist.

PLOS ONE for specific examples.<br><br>**NO authors have competing interests**<br><br>Enter: *The authors have declared that no competing interests exist.*<br><br>**Authors with competing interests**<br><br>Enter competing interest details beginning with this statement:<br><br>*I have read the journal's policy and the authors of this manuscript have the following competing interests: [insert competing interests here]*<br><br>* typeset	
**Ethics Statement**<br><br>Enter an ethics statement for this submission. This statement is required if the study involved:<br><br>• Human participants<br>• Human specimens or tissue<br>• Vertebrate animals or cephalopods<br>• Vertebrate embryos or tissues<br>• Field research<br><br>Write "N/A" if the submission does not require an ethics statement.<br><br>General guidance is provided below. Consult the submission guidelines for detailed instructions. **Make sure that all information entered here is included in the Methods section of the manuscript.**	The Ethics committee of the Shahid Beheshti School of Dentistry approved this study (ethic code: IR.SBMU.RIDS.REC.1394.155). In addition, more ethical concerns have been quoted in the M&M section: "After obtaining the oral consent of the workers for taking part in the study, they have given explanation on how to fill the questionnaire, the confidentiality of their answers, and the possibility of withdrawing from the study at each stage. They were then asked to answer the questionnaires carefully and according to the relevant instructions. The questionnaire was read to illiterate workers and their answers were recorded by one of the authors (RD)."

**Format for specific study types**<br><br>**Human Subject Research (involving human participants and/or tissue)**<br>• Give the name of the institutional review board or ethics committee that approved the study<br>• Include the approval number and/or a statement indicating approval of this research<br>• Indicate the form of consent obtained (written/oral) or the reason that consent was not obtained (e.g. the data were analyzed anonymously)<br><br>**Animal Research (involving vertebrate animals, embryos or tissues)**<br>• Provide the name of the Institutional Animal Care and Use Committee (IACUC) or other relevant ethics board that reviewed the study protocol, and indicate whether they approved this research or granted a formal waiver of ethical approval<br>• Include an approval number if one was obtained<br>• If the study involved *non-human primates*, add *additional details* about animal welfare and steps taken to ameliorate suffering<br>• If anesthesia, euthanasia, or any kind of animal sacrifice is part of the study, include briefly which substances and/or methods were applied<br><br>**Field Research**<br><br>Include the following details if this study involves the collection of plant, animal, or other materials from a natural setting:<br>• Field permit number<br>• Name of the institution or relevant body that granted permission	
**Data Availability**<br><br>Authors are required to make all data underlying the findings described fully available, without restriction, and from the time of publication. PLOS allows rare exceptions to address legal and ethical concerns. See the PLOS Data Policy and FAQ for detailed information.	No - some restrictions will apply

A Data Availability Statement describing where the data can be found is required at submission. Your answers to this question constitute the Data Availability Statement and **will be published in the article**, if accepted.<br><br>**Important:** Stating ‘data available on request from the author’ is not sufficient. If your data are only available upon request, select ‘No’ for the first question and explain your exceptional situation in the text box.<br><br>Do the authors confirm that all data underlying the findings described in their manuscript are fully available without restriction?	
**Describe where the data may be found in full sentences. If you are copying our sample text, replace any instances of XXX with the appropriate details.**<br><br>• If the data are **held or will be held in a public repository**, include URLs, accession numbers or DOIs. If this information will only be available after acceptance, indicate this by ticking the box below. For example: *All XXX files are available from the XXX database (accession number(s) XXX, XXX.).*<br>• If the data are all contained **within the manuscript and/or Supporting Information files**, enter the following: *All relevant data are within the manuscript and its Supporting Information files.*<br>• If neither of these applies but you are able to provide **details of access elsewhere**, with or without limitations, please do so. For example:<br><br>*Data cannot be shared publicly because of [XXX]. Data are available from the XXX Institutional Data Access / Ethics Committee (contact via XXX) for researchers who meet the criteria for access to confidential data.*<br><br>*The data underlying the results presented in the study are available from (include the name of the third party*	All relevant data are within the manuscript and its Supporting Information files.

and contact information or URL).<br>• This text is appropriate if the data are owned by a third party and authors do not have permission to share the data.<br><br>* typeset	
Additional data availability information:	Tick here if your circumstances are not covered by the questions above and you need the journal's help to make your data available.

Manuscript
Click here to access/download;Manuscript;complete manuscript siman 031200.docx

The relationship between oral health and occupational condition in workers of a cement factory

Running head: Oral health & occupational condition

Hadi Ghasemi*, Reza Darmohammadi, Mahshid Namdari, Zahra Ghorbani

Department of Community Oral Health, School of Dentistry, Shahid Beheshti University of Medical Sciences, Tehran, Iran

Key words: Dental public health, competency-based education, workforce education

*Corresponding author:

Hadi Ghasemi

Department of Community Oral Health, School of Dentistry, Shahid Beheshti University of Medical Sciences, Daneshjou Blvd., Evin

Tehran, Iran

Postal Code: 1983963113

Tel: +98 21 22421813

Fax: +98 21 22421813

email: ha.ghasemi@sbmu.ac.ir, hadighasemi558@yahoo.com

Abstract

Background and aim: Working time outside routine daily working hours is known as shift working. Studies have shown adverse effects of shift working such as stress, obesity, and diabetes on the workers' health. The aim of the present study is to evaluate the association between oral health and occupational condition in shift workers and non-shift workers of a cement factory in Shahroud, Iran.

Methods: Study population comprised 180 male workers of Shahrood cement factory in the year 2015. Convenience sampling was used to select the subjects and continued until completing 90 in each group of shift- and non-shift workers. Data collection included WHO standard oral health questionnaire, health and safety executive (HSE) questionnaires, together with clinical oral examination. The Chi-square test, Pearson correlation coefficient, and generalized Poisson model were employed for statistical evaluation.

Results: Mean age of the workers was 39.19 (±9.48); 53% had educational level of less than diploma. Their mean DMFT was 12.89 (±5.75) which correlated with number of years in shift work schedule (Pearson correlation coefficient: 0.41; $p<0.001$) but not correlated with job stress (Pearson correlation coefficient: -0.11; $p=0.12$). Mean number of deep periodontal pockets among the workers was 5.03 (±1.84) that showed correlation with number of years in shift work schedule (Pearson correlation coefficient: 0.33; $p<0.001$) but no correlation with job stress (Pearson correlation coefficient: -0.03; $p=0.68$). Adherence to various oral health behaviors was reported by less than half of the workers.

Conclusion: Positive correlation between the prevalence of two main oral diseases and shift working partly signals negative impact of working conditions on oral health among this group of

workers. This calls for oral health promotion programs with modifications in their working environment to facilitate health practices.

Keywords: Shift work schedule, oral health, occupational stress, DMF index, periodontal index

Introduction

Occupation may influence physical and mental health of individuals depending on the type of work and its conditions (1). Special attention has been paid to the relationship between working hours and the amount of job stress with individuals' health. There is a direct relationship between working hours and damage to health (2). In addition to the amount, the distribution of working hours also affects health. The effect of shift work schedule on health has been documented (3). Studies have shown relationship between working in different shifts with several unhealthy conditions such as diabetes(4), cardio-vascular diseases(5), obesity(6), and metabolic syndrome(7). Job stress is another factor that affects health both physically and mentally (8).

Occupation can affect oral health through stress or systemic illnesses caused by overwork (9, 9.5). Scalco et al. assessing (oral) health of 3253 office workers reported that overwork was associated with poorer oral health status and restricted individuals from adherence to oral health-related behaviors(10). Youshino et al. found that frequent stomatitis, gingival swelling and recession, unusual feeling in the mouth, bad breath, clicking sound in the jaw, and worn down teeth were associated with job stress among a group of workers in Japan(11). In Iran, studies regarding the relationship between work conditions and oral health status of workers are,

however, limited. The aim of this study was to evaluate the working conditions including shift work schedule and job stress on oral health in a group of workers of Shahroud Cement Factory.

Methods

The target population for this cross-sectional study was 180 male workers of Shahroud Cement Factory in the year 2015. They were selected as a convenience sample from total 500 workers of the factory. Data gathering continued until reaching 90 workers from each of the shift work schedule or not shift work schedule (stable shift) in a period of twenty consecutive working days. Data collection included questionnaire and clinical oral examination. Questionnaire survey consisted of two sections: 1. Health, oral health, quality of life, and work related questions and 2. Job stress questions.

The first section of the questionnaire was prepared in simple language and in accordance with the level of knowledge of the studied workers and was finalized after evaluating its validity and Reliability. To determine the validity, the questionnaire was given to 15 workers. After completing the questionnaire, they reported that the questionnaire was simple to understand, appropriate with no ambiguity or inadequacy in the meanings of the words. In order to determine the reliability of the questionnaire, the test-retest method was used. The questionnaire completed by 15 workers two times with two weeks interval. The reliability coefficient obtained at this stage was 0.7, which is in an acceptable range.

This section of the questionnaire included questions on the workers' demographic information such as age, years of work experience, shift work schedule (yes or no), having a second job (yes or no), level of education, oral health related behaviors (frequency of toothbrushing, time of last

dental visit, frequency of consumption of sugary snacks and drinks), aspects of oral health-related quality of life (frequency of dental pain, restriction of eating and communication with others due to oral problems).

The second section of the questionnaire included the HSE (Health and Safety Executive) questionnaire(12) which employed to assess job stress. This questionnaire includes 35 questions in seven sub-areas as follows: 1. Demand: issues such as workload, characteristics and work environment, 2. Control: to what extent individuals can be said to be doing their job, 3. Support of the officials: the amount of support that a person receives from his management and service institution, 4. Peer support: the amount of support that individuals receives from their colleagues, 5. Relationships: “includes promoting positive working to avoid conflict and dealing with unacceptable behavior”, 6. Role: “whether people understand their role within the organization and whether the organization ensures that the person does not have conflicting roles”, and finally 7. Change: “how organizational change is managed and communicated”. The validity and reliability of the Persian version of this questionnaire has been proven in the Iranian population(13).

The answer to each question was based on a 5-point Likert scale from never (score 1) to always (score 5), with higher scores indicating higher job stress. Based on total score (theoretical range: 35-175), the participants were divided into two groups with low (<115) and high (≥116) job stress.

The Ethics committee of the Shahid Beheshti School of Dentistry approved this study (ethic code: IR.SBMU.RIDS.REC.1394.155). After obtaining the oral consent of the workers for taking part in the study, they have given explanation on how to fill the questionnaire, the confidentiality

of their answers, and the possibility of withdrawing from the study at each stage. They were then asked to answer the questionnaires carefully and according to the relevant instructions. The questionnaire was read to illiterate workers and their answers were recorded by one of the authors (RD).

Clinical oral examination included measuring periodontal pocket depth and DMFT index based on the WHO method for oral health survey(14) which was performed by one of the authors (RD). Periodontal pocket with >4 mm depth was regarded as sign of periodontal disease.

The Chi-square test, Pearson correlation coefficient, and generalized Poisson model served as statistical evaluation.

Results

The workers' mean age was 39.19 (±9.48) with average 13.91 (±7.24) years of work experience and 64% reported <10 years in shift work schedule. As it appears in Table 1, majority of the workers reported that working in the cement factory is their only job and for more than half of the workers, education level was below diploma and high perceived job stress.

Table 1. Distribution (%) of the workers (n=180), based on their background and working factors.					
		All (%)	Years in shift work schedule		
			< 10 (%)	≥ 10 (%)	P-value*
Age (years)	≤ 35	62 (34)	54(47)	8 (13)	<0.001
	36-50	97 (54)	56 (48)	41 (64)	
	≥ 51	21 (12)	6 (5)	15 (23)	
Working experience (years)	< 15	93 (52)	74 (64)	19 (30)	<0.001
	≥ 15	87 (48)	42 (36)	45 (70)	
Having second job	No	155 (86)	102 (88)	53 (83)	0.37
	Yes	25 (14)	14 (12)	11 (17)	
Education level	< Diploma	96 (53)	59 (51)	37 (58)	0.43
	≥ Diploma	84 (47)	57 (49)	27 (42)	
Job stress level	Low	86 (48)	54 (47)	32 (50)	0.75
	High	94 (52)	62 (53)	32 (50)	
* Statistical evaluation by the Chi-square test.					

Table 2 shows distribution of the workers' oral health-related behaviors and aspects of oral health –related quality of life according to their shift work pattern. The respondents' adherence to the oral health-related behaviors was as follows: at least twice per day toothbrushing by 9% (at least once per day by 18%), using dental floss at least once per day by 1%, eating or drinking sugary foods at least once per day by 42%, and having a dental visit in the past year by 33%. More than 80% of the respondents reported that they had rarely or never pain, difficulty in communication, and difficulty in eating due to dental problems in the past three months.

As it appears in table 2, those workers with <10 years of shift work schedule reported higher frequency of at least twice per day toothbrushing (p=0.05), less frequency regarding difficulty in communication (p<0.001), and eating (p=0.02) due to dental problems in the past three months. Having a dental visit in the past year was more prevalent among the workers with ≥10 years in shift work schedule.

Table 2. Distribution (%) of the workers' (n=180) oral health-related behaviors and quality of life.

		All (%)	Years in shift work schedule		
			< 10 (%)	≥ 10 (%)	P-value[1]
Toothbrushing	≥2/day	16 (9)	14 (12)	2 (3)	0.05
	<2/day	164 (91)	102 (88)	62 (97)	
Using dental floss	≥1/day	2 (1)	2 (2)	0 (0)	0.54
	<1/day	178 (99)	114 (98)	64 (100)	
Dental visit in the past 12 months	Yes	60 (33)	32 (28)	28 (44)	0.03
	No	120 (67)	84 (82)	36 (56)	
Sugary snack or drink between main meals	≤1	75 (42)	55 (43)	25 (39)	0.63
	>1	105 (58)	66 (57)	39 (61)	
Dental pain in the past 3 months	Seldom[2]	141 (78)	96 (83)	45 (70)	0.06
	Regularly	39 (22)	20 (17)	19 (30)	
Difficulty in communication due to dental problems in the past 3 months	Seldom	164 (91)	114 (98)	50 (78)	<0.001
	Regularly	16 (9)	2 (2)	14 (22)	
Difficulty in eating due to dental problems in the past 3 months	Seldom	145 (81)	100 (86)	45 (70)	0.02
	Regularly	35 (19)	16 (14)	19 (30)	

1. Statistical evaluation by the Chi-square test.
2. Seldom: rarely or never; regularly: sometimes, mostly or always

Mean of DMFT among the workers was 12.89 (±5.75), that showed a correlation with number of years in shift work schedule (Pearson correlation coefficient: 0.41; p<0.001) but no correlation with job stress (Pearson correlation coefficient: -0.11; p=0.12). Mean number of periodontal pockets with >4mm depth among the participants of this study was 5.03 (±1.84). This variable also showed a correlation with number of years in shift work schedule (Pearson correlation coefficient: 0.33; p<0.001) but no correlation with job stress (Pearson correlation coefficient: -0.03; p=0.68).

Table 3. Determinants of dental caries and periodontal disease among the workers (n=180)*.

	IRR	SE	*p*-value	95% CI
**DMFT**				
Shift work schedule (0=no, 1=yes)	1.003	0.003	0.399	0.99-1.01
Age (years)	1.027	0.002	<0.001	1.02-1.03
Education level (0=<Diploma, 1=≥ Diploma)	1.037	0.047	0.428	0.94-1.13
Job stress score	0.999	0.002	0.805	0.99-1.00
Have a second job (0=no, 1=yes)	1.066	0.069	0.327	0.93-1.21
Frequency of dental visit in the past year	0.972	0.022	0.218	0.93-1.01
Frequency of tooth brushing	0.940	0.014	**<0.001**	**0.91-0.96**
Frequency of using dental floss	1.118	0.070	0.074	0.98-1.26
Constant	5.432	1.743		
**Number of sites with periodontal pocket >4mm**				
Shift work schedule (0=no, 1=yes)	1.005	0.003	0.141	0.99-1.01
Age (years)	1.011	0.002	<0.001	1.00-1.01
Education level (0=<Diploma, 1=≥ Diploma)	1.029	0.045	0.516	0.94-1.12
Job stress score	1.000	0.002	0.958	0.99-1.00
Have a second job (0=no, 1=yes)	1.051	0.065	0.421	0.93-1.18
Frequency of dental visit in the	0.999	0.022	0.978	0.95-1.04

past year				
Frequency of toothbrushing	0.968	0.013	**0.018**	**0.94-0.99**
Frequency of using dental floss	1.123	0.059	**0.028**	**1.01-1.24**
Constant	2.867	0.893		

*As assessed by two similar generalized Poisson models
IRR: incidence rate ratio, SE: standard error

Table 3 presents factors which were interested to evaluate their effect on the expected count of DMFT and the number of sites with periodontal disease among the workers based on two similar models. Shift work schedule seems to have no impact on the two outcome variables namely DMFT and periodontal pocket. Higher frequency of using dental floss increased the probability of having periodontal pocket >4mm (IRR=1.123, 95% CI=1.01-1.24). Higher frequency of toothbrushing decreased the probability of having more DMFT (IRR=1.066, 95% CI=0.91-0.96) and more periodontal pocket >4mm (IRR=1.033, 95% CI=0.94-0.99).

Discussion

Findings of the present study failed to show a relationship between job stress with shift work schedule and oral health outcomes among the workers. A weak correlation, however, was found between shift work schedule and oral health outcomes. Favorable level of oral health behaviors among the workers was very infrequent especially for the use of dental floss and toothbrushing.

Psychological stress in result of working condition has been suggested as a risk factor for dental caries and periodontal diseases. One of the probable mechanisms in this regard is the effect of psychological stress on the individual's oral health behaviors(15). Among the workers in the

present study, work-related stress showed relationship neither with measures of dental caries and periodontal disease, nor with oral health related behaviors. Studies on the relationship between job stress and measures of oral health, however, show some sort of controversy. While some studies found that higher level of work stress is associated with poor oral health related quality of life (16) (11)., and less favorable level of oral health related behaviors(17), a systematic review found no evidence on the association of work stress with dental caries and tooth loss but some potential association between periodontal disease and work stress (18).

Being in the shift schedule for a long time correlated with less favorable oral health status among the workers in the present study in a way that those workers with more years in shift schedule experienced higher level of dental caries, periodontal diseases, and more difficulty in communication and eating due to dental problems. This finding is in line with studies showing the association of non-standard work schedules with less favorable health outcomes (19) (20, 21). Non-standard working schedule is proposed as a trigger for physiological changes resulting to disturbances in sleep pattern and circadian rhythms and ultimately reduce the workers' adaptive capabilities (22). To the best of our knowledge this study seems to be the first in quest for a relationship between shift working schedule and oral health outcomes. On the other hand, the relationship between various working time characteristics with employees' health status has been studied largely. For example, young adult American workers without a regular day shift pattern of working showed higher risk for a wide range of health-related outcomes comparing to workers with standard regular daytime schedule(20). Irregular work schedule found to have negative impact on quality of sleep and sense of coherence among a group of Hungarian nurses (21). Due to the importance of oral health on the quality of life and productivity of the workers,

incorporating the oral health indicators into the comprehensive studies of the workers' health is called for.

Adherence to healthy behaviors is an essential prerequisite for good oral health status. Very little percentage of the participants in this study, however, reported complying with toothbrushing at least twice per day and using dental floss. Having a dental visit and restricting the sugary food were also a rare finding since about two third of the workers did not report a dental visit and close to 60% reported more than once per day eating sugary foods. This may be a reflection of either underestimation of the importance of best oral hygiene practices by those workers or their difficult working condition that hinder them from performing recommended level of oral health behaviors. Data on the oral health of the workers in Iran is rare. However, findings of a recent study on a group of employees in Tehran (23), show higher prevalence for toothbrushing (28%), using dental floss (48%), and dental visit (77%) compared to the present study. Another study from Mashhad city (24), that included employees as participants, found that at least 34% brush their teeth twice per day and 35% use dental floss in a daily basis. Different figures of the prevalence of oral health behaviors reflect underestimation of the importance of recommended criteria for favorable level of oral health and also different level of socio-economic status and cultural diversity of the participants in the previous studies. Reaching to higher rates of favorable level of oral health behaviors among working age population is not a dream since findings of some studies from foreign countries show promising results in this regards. For example, toothbrushing on at least 2/day basis has been reported by more than 90% of a group of Korean workers (25) and around 80% of a representative sample of adult population from eight European countries (26).

Findings of this study should be interpreted with caution due to some limitations. First is the inherent limitation of cross-sectional study which cannot make a causal inference between variables. Second is the convenient sampling which makes the generalization of the findings to the whole population uncertain. Third is the limitation of questionnaire surveys which is prone to socially acceptable answers and recall bias.

Conclusion

Correlation of shift working with dental caries and periodontal diseases among the workers in this study speaks for a potential risk for oral health of workers. Provision of rather stable working time would help workers for better adherence to optimal oral health behaviors which in turn enhance their overall health status and productivity

Acknowledgements

Declarations

Ethics approval and consent to participate

The Ethics committee of the Shahid Beheshti School of Dentistry approved this study (ethic code: IR.SBMU.RIDS.REC.1394.155). In addition, more ethical concerns have been quoted in the M&M section:"After obtaining the oral consent of the workers for taking part in the study, they have given explanation on how to fill the questionnaire, the confidentiality of their answers, and the possibility of withdrawing from the study at each stage. They were then asked to answer

the questionnaires carefully and according to the relevant instructions. The questionnaire was read to illiterate workers and their answers were recorded by one of the authors (RD)."

Consent for publication

Not applicable

Availability of data and material

Data are available from the authors upon reasonable request and with permission of Dr Hadi Ghasemi.

Competing interests

The authors declare that they have no competing interests.

Funding

No funding

Authors' contributions

HG supervised the whole project from conception the idea, writing the proposal, preparing the questionnaire, supervising the implementation, doing the statistical analysis, and writing the manuscript. RD contributed mainly in execution of the project and data collection. MN mainly contributed to the statistical analysis and also helped in drafting the manuscript. ZG gave consultations in the planning and implementation phases and commented on manuscript drafts. All authors read and approved the final manuscript.

Acknowledgements

We thank all the participating workers for their cooperation.

References

1. Van Uffelen JG, Wong J, Chau JY, Van Der Ploeg HP, Riphagen I, Gilson ND, et al. Occupational sitting and health risks: a systematic review. 2010;39(4):379-88.
2. Berniell I, Bietenbeck JJE, Biology H. The effect of working hours on health. 2020;39:100901.
3. Moreno CR, Marqueze EC, Sargent C, Wright Jr KP, Ferguson SA, Tucker PJIh. Working Time Society consensus statements: Evidence-based effects of shift work on physical and mental health. 2019;57(2):139-57.
4. Hulsegge G, Proper KI, Loef B, Paagman H, Anema JR, van Mechelen WJIAoO, et al. The mediating role of lifestyle in the relationship between shift work, obesity and diabetes. 2021:1-9.
5. Torquati L, Mielke GI, Brown WJ, Kolbe-Alexander TJSjow, environment, health. Shift work and the risk of cardiovascular disease. A systematic review and meta-analysis including dose–response relationship. 2018;44(3):229-38.
6. Sun M, Feng W, Wang F, Li P, Li Z, Li M, et al. Meta-analysis on shift work and risks of specific obesity types. 2018;19(1):28-40.
7. Oh J-I, Yim HWJIh. Association between rotating night shift work and metabolic syndrome in Korean workers: differences between 8-hour and 12-hour rotating shift work. 2018;56(1):40-8.
8. Siegrist J, Wahrendorf M, Siegrist. Work stress and health in a globalized economy: Springer; 2016.
9. Marcenes WS, Sheiham AJSs, medicine. The relationship between work stress and oral health status. 1992;35(12):1511-20.
9.5. Redondo-Flórez L, Fernández-Lucas J, Clemente-Suárez VJ. Cultural differences in stress-related psychological, nutrition, physical activity and oral health factors of professors. Nutrients. 2020 Dec;12(12):3644.
10. Scalco GPdC, Abegg C, Celeste RK, Hökerberg YHM, Faerstein EJC, Coletiva S. Occupational stress and self-perceived oral health in Brazilian adults: a Pro-Saude study. 2013;18:2069-74.
11. Yoshino K, Suzuki S, Ishizuka Y, Takayanagi A, Sugihara N, Kamijyo HJIh. Relationship between job stress and subjective oral health symptoms in male financial workers in Japan. 2016.
12. Cousins* R, Mackay CJ, Clarke SD, Kelly C, Kelly PJ, McCaig RHJW, et al. ‘Management standards’ work-related stress in the UK: Practical development. 2004;18(2):113-36.
13. Azad ME, Gholami FM. Reliability and validity assessment for the HSE job stress questionnaire. 2011.
14. Organization WH. Oral health surveys: basic methods: World Health Organization; 2013.
15. Gomaa N, Glogauer M, Tenenbaum H, Siddiqi A, Quiñonez CJPo. Social-biological interactions in oral disease: a ‘cells to society’view. 2016;11(1):e0146218.
16. Acharya S, Pentapati KCJIdj. Work stress and oral health-related quality of life among Indian information technology workers: an exploratory study. 2012;62(3):132-6.
17. Woo S-H, Ju OJJJodhs. Stress and oral health care in nonhealth-related majors. 2015;15(5):527-35.
18. Sato Y, Saijo Y, Yoshioka EJBo. Work stress and oral conditions: a systematic review of observational studies. 2021;11(5):e046532.

19. Bushnell PT, Colombi A, Caruso CC, Tak SJIh. Work schedules and health behavior outcomes at a large manufacturer. 2010;48(4):395-405.
20. Winkler MR, Mason S, Laska MN, Christoph MJ, Neumark-Sztainer DJS-pH. Does non-standard work mean non-standard health? Exploring links between non-standard work schedules, health behavior, and well-being. 2018;4:135-43.
21. Fusz KSJUoP. Shift Work Schedule Types of Nurses in Hungary and Their Effects on Health Status. 2017.
22. Kervezee L, Kosmadopoulos A, Boivin DBJEJoN. Metabolic and cardiovascular consequences of shift work: The role of circadian disruption and sleep disturbances. 2020;51(1):396-412.
23. Ghasemi H, Sohrabi N, Al-Eshaghi N, Hajabedini A, Khoshnevisan MJJoDS, Shahid Beheshti University of Medical Sciences. Oral Health Status and its Determinants among a Group of Iranian Employees.37(3):77-82.
24. Moeintaghavi A, Arab H, Sargolzaei N, Dorri M, Darvishzadeh F, Alizadeh MJJoDM, et al. Oral Health-Related Quality of Life: A Cross-Sectional Survey among Adult Patients in Mashhad, Iran. 2013;2(4):114-20.
25. NamKoong E-J, Ma D-SJJoKAoOH. The effects of job characteristics and non-regular work on the toothbrushing habit and oral check-up in Korean worker: using data from the 7th Korea National Health and Nutrition Examination Survey (KAHANES, 2016). 2019;43(4):204-9.
26. Nihtila A, West N, Lussi A, Bouchard P, Ottolenghi L, Senekola E, et al., editors. Oral health behavior and lifestyle factors among overweight and non-overweight young adults in Europe: A cross-sectional questionnaire study. Healthcare; 2016: Multidisciplinary Digital Publishing Institute.
